# Corticosterone Preexposure Increases NF-κB Translocation and Sensitizes IL-1β Responses in BV2 Microglia-Like Cells

**DOI:** 10.3389/fimmu.2018.00003

**Published:** 2018-01-22

**Authors:** JiaJun Liu, Sanam Mustafa, Daniel Thomas Barratt, Mark Rowland Hutchinson

**Affiliations:** ^1^Adelaide Medical School, Discipline of Physiology, The University of Adelaide, Adelaide, SA, Australia; ^2^Australian Research Council Centre of Excellence for Nanoscale BioPhotonics, University of Adelaide, Adelaide, SA, Australia; ^3^Adelaide Medical School, Discipline of Pharmacology, The University of Adelaide, Adelaide, SA, Australia

**Keywords:** cytokines, NF-κB, cell morphology, BV2 microglia cells, neuroendocrinology, glucocorticoids, glucocorticoid receptors, IL-1β

## Abstract

Corticosterone (CORT), a critical mediator of the hypothalamus pituitary adrenal axis in rodents, is a stress hormone that is classically viewed as possessing immune-suppressive properties. CORT is now appreciated to also mediate the neuroimmune-priming effect of stress to innate-immune stimulation, and hence serves as a mechanistic link to the neuroimmune involvement in stress-related disorders. However, these dichotomous actions of CORT remain poorly defined. This study investigated the conditions and concentration dependency of CORT’s actions required to prime the innate-immune system. Here, we measured the effect of CORT pretreatment on the downstream pro-inflammatory responses of BV2 mouse microglia-like cells stimulated by lipopolysaccharide (LPS). We quantified the concentration-dependent CORT-mediated attenuation and enhancement of LPS-stimulated inflammatory response. A high physiological concentration (500 nM) of CORT attenuated LPS-induced inflammatory IL-1β cytokine production in a glucocorticoid receptor-dependent manner. However, a low concentration (50 nM) of CORT increased expression and release of IL-1β in a mineralocorticoid receptor-dependent manner, with accompanied increases in NF-κB translocation and changes to related gene transcription. These results suggest that a mild elevation in CORT may cause selective adaptations in microglia-like cells to overrespond to a second immune challenge in a non-classical manner, thus partially explaining both pro- and anti-inflammatory effects of CORT reported in the literature.

## Introduction

### Chronic Stress Can Be Pro-inflammatory

Chronic psychological stress is associated with the development of psychosomatic disorders such as posttraumatic stress disorder and major depressive disorder (1, 2), as well as the exacerbation and persistence of neurological damage (3, 4). Yet, while the neuroendocrine responses to stress are well established, the inflammatory nature of the conditions suggests that immune dysregulation plays an integral role in chronic stress.

Chronic stress itself can result in increased pro-inflammatory innate-immune responses. For example, blood monocytes of non-professional patient caregivers were found to produce more IL-6 in response to lipopolysaccharide (LPS) stimulation (5). Moreover, physically healthy soldiers with combat experience also exhibited increased plasma C-reactive-protein (CRP), an acute-phase protein produced during inflammation, which further correlated with depressive and posttraumatic stress disorder assessment scores (2). Meta-analyses have further uncovered a link between physiological stress-related depression and increases in pro-inflammatory factors such as CRP, IL-1, and IL-6, thus implicating an immune consequence of the physiological stress response (6, 7).

In animal studies, increased pro-inflammatory markers have been observed across several stress paradigms. For example, concurrent neuroendocrine and immune activation can be observed in the peripheral circulation of animals that have received foot shock stress, illustrating the interconnected nature between the neuroendocrine and immune system in stress responses (8, 9). This study also demonstrated that the administration of neuroendocrine hormones, adrenocorticotropic hormone, and corticosteroid-releasing hormone is sufficient to stimulate increased inflammatory markers, *Il6* and *COX2* mRNA expression, in the adrenals of the same animals. Social defeat stress models have also elicited increased monocyte infiltration across the blood brain barrier to specific brain sites (9), while also increasing microglial *Ccl2* and *Il1b* mRNA expression (10). In addition, prenatal restraint stress (11) and chronic mild stress both induced increased microglial reactivity (12). Taken together, stress can result in an inflammatory event *in vivo*.

### Microglia As a Key Stress Sensitive Cell Type

Microglia, the resident immunocompetent cells of the CNS, have become central to the investigation of stress effects on inflammation. An increase in hypothalamic IL-1β cytokine as a result of foot shock stress can be blocked by minocycline, a glial attenuator (13). More recently, stress has been shown to sensitize hippocampal microglia reactivity to LPS stimulation, resulting in increased *Il1b* mRNA (14). This effect was attenuated using a glucocorticoid receptor (GR) antagonist, thus demonstrating that glucocorticoids [corticosterone (CORT)] (the end product of the neuroendocrine stress response) can modify microglial function, causing a “primed” state to further immune challenges. Their studies have also highlighted the role of the NLRP3 inflammasome in the inflammatory actions of stress. The NLRP3 inflammasome acts to cleave pro-IL-1β into the mature form before its release (15). The increased sensitivity of microglia to inflammatory signals could have detrimental consequences for various neurodegenerative diseases (4, 16).

### Neuroendocrine Stress Response and Innate-Immune Function

The implication of glucocorticoids in the immune-priming effect is paradoxical, since CORT is classically regarded as strongly immune suppressive (17–19). However, there is some evidence that the timing of the immune challenge is important toward glucocorticoid actions. It has been hypothesized that CORT is anti-inflammatory in the acute phase during stressor onset but can sensitize the immune system during the “recuperation phase” after the stressor has been resolved (20). This effect could also be in conjunction with alterations to the innate-immune system, most notably the TLR4 pathway that is capable of responding to endogenous and exogenous factors, or the inflammasome pathway that is necessary for the production and release of IL-1β and IL-18 (1). However, direct actions of CORT in this priming effect on microglia are not well understood.

Two main hypotheses have emerged as explanations for the immune-stimulatory effect of CORT, mediated *via* the two main CORT-binding steroid receptors GR and mineralocorticoid receptor (MR), respectively (21). MR activity has been shown to induce pro-inflammatory outcomes in BV2 microglia-like cells, measured *via* increased TNF-α and IL-6 gene transcription (22). Conversely, although GR signaling causes immunosuppression, the prolonged activation of GR can induce a state of “glucocorticoid resistance,” which has been demonstrated to increase p65 NF-κB DNA-binding activity (23), and induce epigeneti adaptations *via* inhibition of histone deacetylase 2 expression (24, 25). However, the exact role of GR and MR in the context of immune priming is presently unclear.

To test the hypothesis that CORT can be anti-inflammatory during ongoing exposure but can leave the immune system sensitized after its removal, this study aimed to investigate the pro- and anti-inflammatory actions of CORT in microglia-like BV2 cells, focusing on the IL-1β release pathway following NF-κB activation through administration of LPS and TNF-α as innate-immune stimulants. In addition, the conversion and release of IL-1β was also investigated. Finally, GR and MR dependency of CORT effects was assessed using specific antagonists to each receptor.

## Materials and Methods

### Cell Culture

BV2 microglia-like cell lines were maintained in Dulbecco’s modified Eagle’s medium supplemented with 10% (v/v) fetal bovine serum and 2 mM l-glutamine + 50 U/ml penicillin + 50 µg/ml streptomycin + 100 µg/ml Normocin. Cells were grown in a humidified incubator of 95% air/5% CO_2_ at 37°C. BV2 cells were plated at a density of 7.5 × 10^4^ cells/well in 24-well plates for cytokine experiments, 5 × 10^4^ cells/well in 12-well plates for fluorescent immunocytochemistry analysis, and 2 × 10^5^ cells/well in 6-well plates for gene expression studies.

### Experimental Design and *In Vitro* Cell Treatment Timing

**Figure 1:**
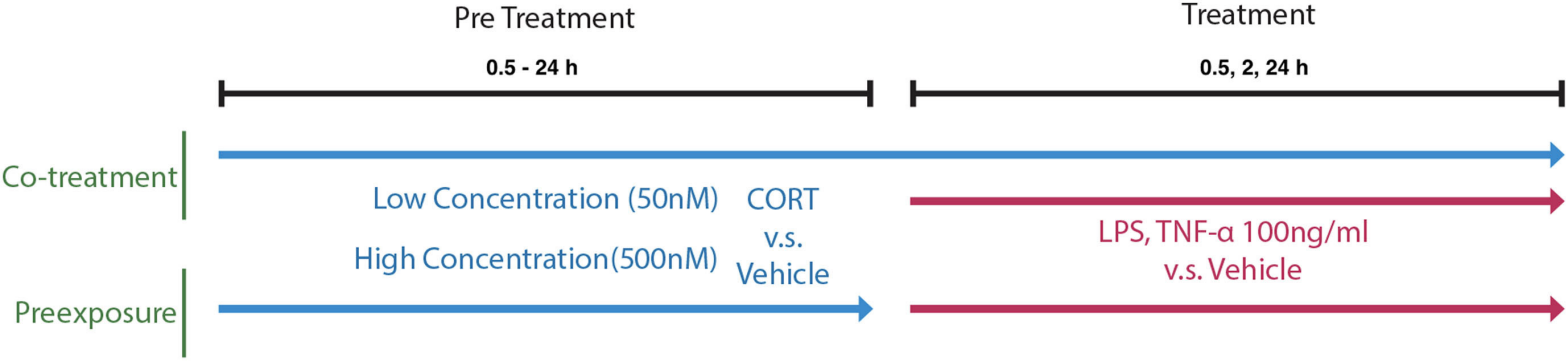
A schematic of the *in vitro* cell treatment timing of preexposure and co-treatment paradigms.

A concentration response was characterized using BV2 cells pretreated with 50 nM–1 µM CORT or volume-matched ethanol vehicle for 24 h before LPS (100 ng/ml) or vehicle treatment conditions. Pretreatment was either left present during immune stimulation (co-treatment model) or removed before immune stimulation (preexposure model) and supernatant IL-1β was measured. The selection of the 24 h time point for CORT pretreatment at the concentration of 50 nM was further verified in a time response (0.5–24 h). A 24 h administration of 50 and 500 nM of CORT pretreatment was selected for further experiments measuring 100 ng/ml TNF-α (R&D Systems, 410-MT/CF, USA)-induced supernatant IL-1β release, as well as LPS-induced NF-κB translocation. Concurrent IL-1β release with intracellular protein expression was also measured after 24 h LPS treatment, while gene transcription was measured after 6 and 24 h of LPS administration (Figure 1).

Since CORT can signal *via* both GR and MR, respective specific inhibitors mifepristone and spironolactone were co-incubated with CORT pretreatment conditions at a concentration of 1 µM. This concentration has been verified in previous studies using BV2 cells (22, 26). Furthermore, caspase-1 and -4 dependency of LPS-induced IL-1β responses was tested using putative inhibitor z-YVAD-FMK (R&D Systems, FMK005, USA), which was co-incubated with LPS treatment at concentrations between 100 nM and 10 µM. A 5 µM concentration of z-YVAD-FMK has previously been shown to inhibit IL-1β release from BV2 cells (27). Collected supernatants were centrifuged at 500 × *g* for 10 min at 4°C, and cells were harvested to prepare cell lysates for further western blot or qPCR analysis. All treatments were paired with respective concentration-matched vehicle controls within each biological replicate.

### Protein and Released Cytokine Quantification

Intracellular NLRP3 and pro-IL-1β were quantified using western blot analysis. Following treatment, adherent cells were washed three times in 1 ml ice-cold PBS, then incubated on ice with 100 µl RIPA buffer for 5 min. Cell lysates were transferred to 1.5 ml tubes, rotated on a tube rotator at 4°C for 1 h, and subsequently centrifuged at 20,000 × *g* at 4°C for 10 min to remove cellular debris. Protein concentration was determined (BCA assay, Thermo Fisher 23225) and normalized to 1 µg/µl in laemmli buffer and stored at −80°C before analysis by SDS-PAGE and western blotting. Twenty-five micrograms of total protein were resolved on a 4–12% Bis–Tris gel at a constant 200 V and transferred onto nitrocellulose membranes at 20 V. Membranes were blocked for 2 h at room temperature in 5% skim milk in TBS-Tween 20 and incubated overnight at 4°C in primary antibodies for NLRP3 (adipogen AG-20B-0014, 1:2,000), pro-IL-1β (Abcam 9722, 1:1,000), and loading control Beta Actin (Sigma-Aldrich A2066, 1:2,000). Appropriate secondary antibodies (1:10,000 dilution) conjugated to either 700 or 800 nm infrared fluorophores were applied for 1 h at room temperature, and blots were developed using the Odyssey scanner. Western blot image analysis was performed using ImageJ (64-bit) software.

Released cytokines were measured using ELISA for IL-1β (BD bioscience 559603) and IL-6 (BD bioscience 555240) in half-area 96-well plates according to the manufacturer’s specifications. Absorbance wavelengths (405 nm signal–560 nm signal correction), indicating cytokine levels, were measured using a Synergy MX plate reader (BioTek). IL-1β and IL-6 levels were quantified against known concentrations of IL-1β standards between 15.625 and 2,000 pg/ml and between 15.625 and 1,000 pg/ml, respectively. Assay acceptance criteria were determined by an *R*^2^ value larger than 0.95 for standard curve fit.

### Nuclear Translocation of NF-κB and Changes in Cell Morphology

NF-κB is a transcription factor downstream of innate-immune receptor activation and is usually bound to Ikkb in the cytoplasm. The p65 subunit translocation to the nucleus is indicative of increased transcription of pro-inflammatory genes and therefore is a measure of the innate-immune response [see Ref. (28), for a review]. BV2 cells display NF-κB/p65 nuclear translocation in response to LPS exposure (29).

Intracellular localization of NF-κB was quantified using fluorescent immunocytochemistry. Briefly, BV2 cells were plated onto poly-d-lysine coated coverslips. BV2 cells underwent either low (50 nM) or high (500 nM) concentration CORT or vehicle preexposure for 24 h before replacement with 1 μg/ml LPS or vehicle for a further 30 min or 2 h. Cells were fixed in 4% PFA with 5% sucrose for 10 min and stained with the respective antibodies (Rabbit anti-NF-κB, 1:500, Abcam ab 16502) for 2 h. Secondary antibodies (Donkey anti-Rabbit 488, 1:1,000 dilution, Life Technologies A21206; 488 nm excitation), DAPI (405 nm excitation, 1:10,000; nuclear stain), and wheat germ agglutin (WGA; 633 nm excitation, 1:200; membrane stain) were then added for a further 1 h. Following staining, coverslips were inverted onto slides and imaged under the confocal microscope (Leica SP-5).

Nuclear translocation of NF-κB was quantified *via* measuring the intensity values of DAPI (nucleus), NF-κB, and WGA (cell membrane) across the longest diameter of the cell, yielding an intensity profile plot for each cell. This was applied to 15 cells per image using FIJI’s distribution of ImageJ-64 bit (30). NF-κB expression was measured by integrating the area under curve using the trapezoid method between the bounds of the nucleus, and between the bounds of the cell membrane. The proportion of NF-κB expression in the nucleus vs total was further normalized to the proportion of nucleus diameter to total cell diameter, thus describing the distribution of NF-κB within the cell:

$$\text{Nuclear Translocation}=\frac{\text{NF-}\kappa \text{B expression within nucleus}}{\text{Total cell NF-}\kappa \text{B}}÷\frac{\text{Nucleus Diameter}}{\text{Cell Diameter}},$$

where Nuclear Translocation degree = 1 would indicate an even distribution of NF-κB between nucleus and cytoplasm, <1 would indicate distribution of NF-κB favoring the cytoplasm, and >1 indicates NF-κB distribution favoring the nucleus.

BV2 cells can present with morphological changes, which can be associated with phagocytic capacity (31). Furthermore, since microglia retract their processes and become amoeboid shaped in response to LPS (32), we thus measured the morphological change in BV2 cells in response to LPS following 50 nM CORT preexposure. Due to the rod-shaped morphology of BV2 cells, cell shape was inferred by measuring the change in proportion of nucleus diameter to cell diameter across the widest width of each cell. For example, a retraction of cell processes, reflected by a reduction in total cell diameter, and an increase in proportion of nucleus to cell diameter, implies an amoeboid shape associated with a pro-inflammatory phenotype (32, 33).

### Statistical Analysis

To assess CORT pretreatment concentration and time effects, IL-1β concentration, measured from supernatant samples, was converted to a percent change from volume and time-matched vehicle pretreated controls. Subsequently, each CORT pretreated group varying in concentration, pretreatment model or time, was compared with baseline value using a linear model with the intercept set at 1× fold.

To quantify effects of treatment (LPS vs vehicle), pretreatment drug (CORT vs vehicle), pretreatment concentration (50 vs 500 nM), and pretreatment model (preexposure vs co-treatment), a four-way ANOVA followed by *post hoc* pairwise comparisons using Tukey’s correction were applied to evaluate total intracellular NLRP3 expression.

To investigate the possible relationship between pro- and released IL-1β, a linear-mixed effects (LME) model was fit to (LPS–vehicle) supernatant IL-1β (sIL1resp) and log([LPS/vehicle] pro-IL-1β) expression (proIL1fold) using the following formula in the nlme package for R:

$$\begin{array}{c} \text{lme}(\text{sIL1resp}\sim \text{log}(\text{proIL1fold}{)}^{⋆}\;{\text{Pre-treatment}}^{⋆}\\ \;\text{Pre-treatment Concentration, random}=(\sim 1|N)), \end{array}$$

where each biological replicate (*N*) was denoted as a random effect to statistically control for repeated measures within each biological replicate. LME models were also used to assess pretreatment effects in TNF-α-induced IL-1β release, as well as GR and MR antagonist effects on CORT pretreatment in respect to IL-1β secretion after LPS treatment. All analysis was done using R (64-bit) statistical program (version 3.3.1) (34).

## Results

### Concentration, Time, and Receptor Dependency of CORT Pretreatment Models on LPS-Induced IL-1β and IL-6 Responses

Lipopolysaccharide treatment (100 ng/ml, 24 h) resulted in an increase in extracellular cytokine concentrations. The mean ± SEM of the measured IL-6 and IL-1β responses were 8.82 ± 1.80 ng/ml and 51.6 ± 3.9 pg/ml, respectively.

#### IL-1β

**Figure 2:**
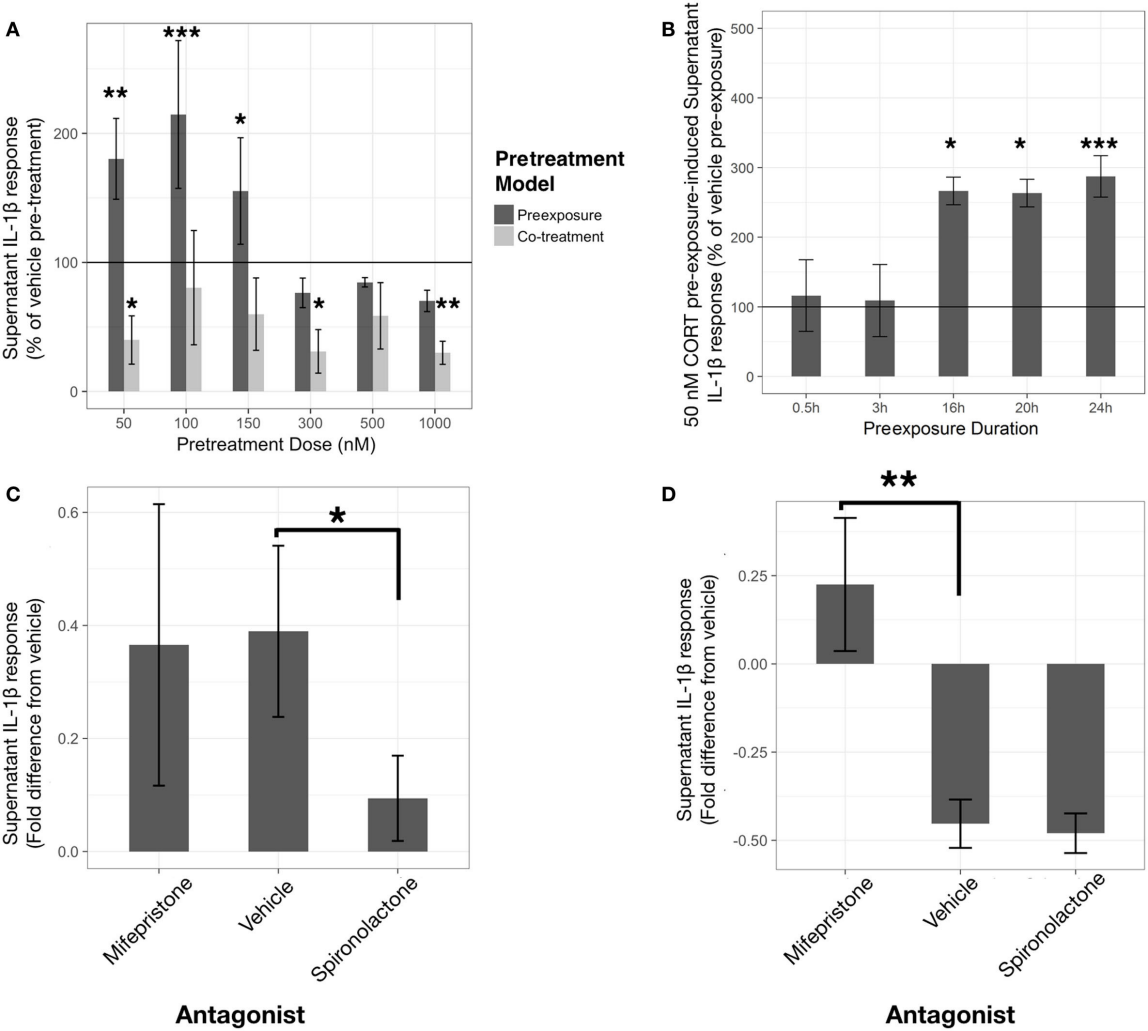
Corticosterone (CORT) preexposure exhibits concentration and time-dependent inhibition and priming of lipopolysaccharide (LPS)-induced IL-1β release in BV2 cells. Low concentration preexposure accentuation of IL-1β release is attenuated by a mineralocorticoid receptor (MR) antagonist, while co-treatment inhibition of IL-1β is reversed by a glucocorticoid receptor (GR) antagonist. **(A)** IL-1β concentration response in 24 h CORT preexposure/co-treatment models measured after 24 h LPS-vehicle treatment (*N* = 6). **(B)** 24 h LPS-induced IL-1β response after 50 nM CORT preexposure varying in length of time (*N* = 4). **(C,D)** Role of GR and MR using specific antagonists, such as mifepristone and spironolactone, respectively, on low concentration CORT preexposure effects on IL-1β release from BV2 cells [**(C)**, *N* = 9], and with co-treatment [**(D)**, *N* = 5]. All supernatant IL-1β values represented as fold difference in LPS-induced IL-1β from vehicle pretreated controls. Error bars represent mean ± SEM. Asterisks denote *p*-values *<0.05, **<0.01, ***<0.001, and ****<0.0001.

Corticosterone preexposure exhibited a biphasic concentration response on LPS-induced supernatant IL-1β (Figure 2A). Low concentration (50 nM) preexposure requires >16 h duration to achieve significant priming of released IL-1β response (Figure 2B).

A linear model with significantly accounted for variation in percentage change of IL-1β release between CORT pretreatment and volume-matched vehicles across the concentration response (*R*^2^ = 0.52, *p* < 0.0001). In addition, the preexposure and co-treatment models elicited significantly different LPS-induced IL-1β release [mean difference = 140%, *t*(36) = 3.91, *p* < 0.001]. Comparisons with vehicle reference group found that co-treatment of CORT with LPS was significantly inhibitory toward IL-1β release at 50 nM [mean difference= −60%, *t*(36) = −2.37, *p* < 0.05], 300 nM [mean difference= −69%, *t*(36) = −2.72, *p* < 0.05], and 1 µM [mean difference= −70%, *t*(36) = −2.76, *p* < 0.01]. Conversely, CORT preexposure demonstrated significant elevation of LPS-induced extracellular IL-1β at 50 nM [mean difference= 80%, *t*(36) = 3.16, *p* < 0.01], 100 nM [mean difference= 115%, *t*(36) = 4.52, *p* < 0.001], and 150 nM [mean difference= 55%, *t*(36) = 2.18, *p* < 0.05] preexposure concentrations.

A linear model assessing the time-dependency of IL-1β release represented as percentage change between 50 nM CORT preexposure and time-matched vehicles was constructed (*R*^2^ = 0.69, *p* < 0.001). When comparing 24 h LPS-induced IL-1β in each preexposure duration to vehicle, CORT only caused significant elevation in IL-1β after 16 h [mean difference= 166%, *t*(13) = 2.96, *p* < 0.05], 20 h [mean difference= 163%, *t*(13) = 2.91, *p* < 0.05], and 24 h [mean difference= 187%, *t*(13) = 5.27, *p* < 0.001].

Previous rodent experiments conducted in the lab have found approximately 500 nM–1 µM CORT concentrations measured from mouse serum following acute stress, while microdialysis of the rodent brain has revealed normal physiological CORT levels ranging between 50 and 100 nM (35). Taken together with the concentration and time responses, further analysis focused on investigation of a low (50 nM) and physiological high (500 nM) concentration for 24 h.

To further investigate the mechanism by which low concentration CORT pretreatment models act to sensitize, or inhibit, the IL-1β response to LPS, specific antagonists to MR and GR, spironolactone (1 μM) and mifepristone (1 μM) respectively, were coadministered with CORT pretreatment conditions (Figures 2C,D). In this experiment, low concentration CORT preexposure and LPS treatment significantly elevated IL-1β measured in the supernatant compared with vehicle controls [*B* = 6.95, *t*(16) = 3.06, *p* < 0.01] (Figure 2C). Spironolactone abolished the priming effect of low concentration CORT preexposure [*B* = −6.06, *t*(16) = −2.14, *p* < 0.05], whereas mifepristone did not significantly modify CORT-induced priming [*B* = −2.43, *t*(16) = −0.86, *p* = 0.40]. On the other hand, LPS-induced IL-1β secretion was significantly inhibited when CORT was co-incubated with LPS treatment [*B* = −11.97, *t*(8) = −4.14, *p* < 0.01]. This CORT co-treatment resultant inhibition effect was abolished by mifepristone [*B* = 14.74, *t*(8) = 3.60, *p* < 0.01] but was not significantly influenced by spironolactone coadministration [*B* = −1.05, *t*(8) = −0.26, *p* = 0.80] (Figure 2D).

#### IL-6

**Figure 3:**
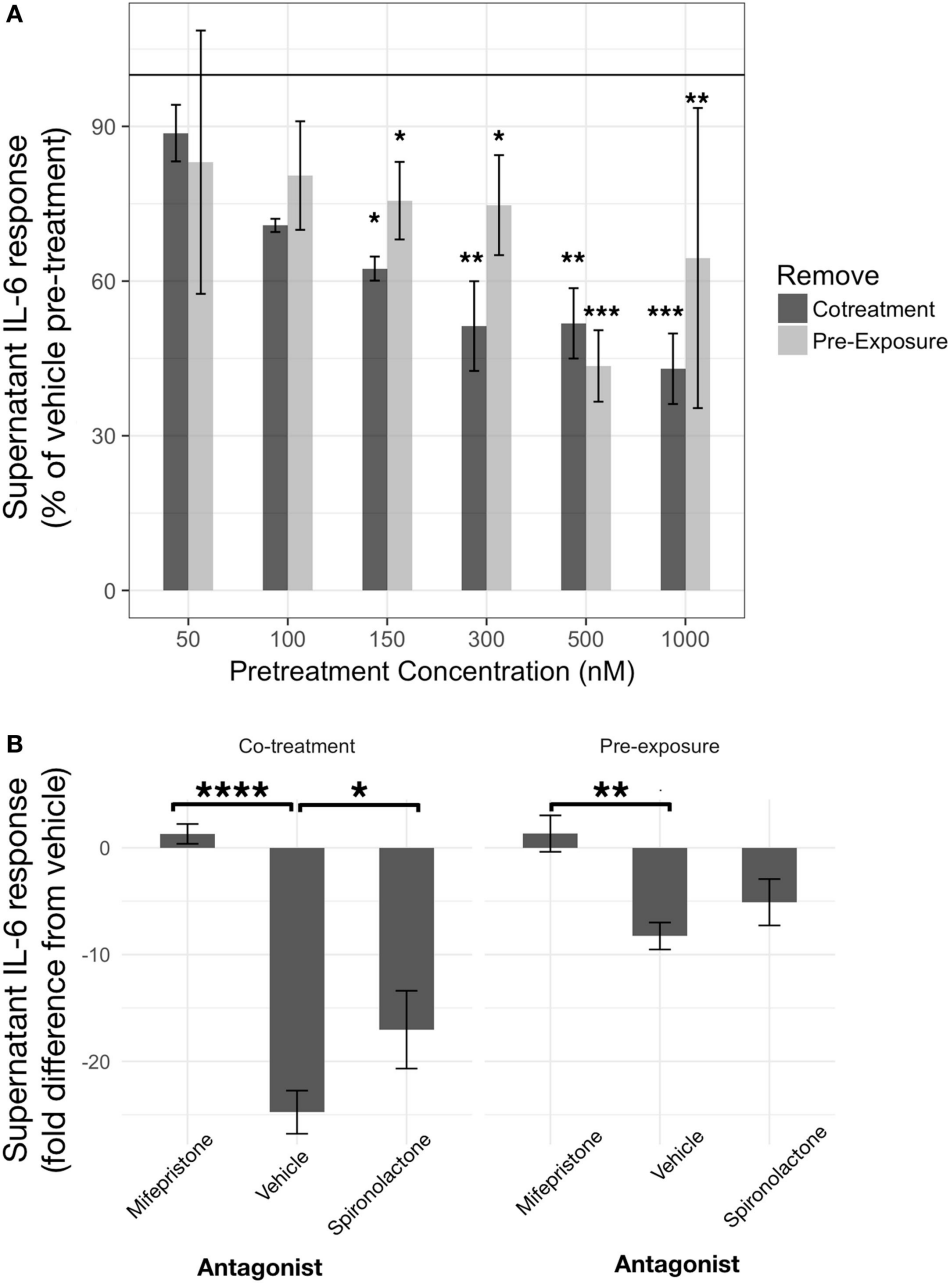
Corticosterone (CORT) pretreatment is inhibitory toward IL-6 release from BV2 cells in a concentration- and glucocorticoid receptor (GR)-dependent manner. **(A)** IL-6 response measured after 24 h CORT pretreatment and 24 h lipopolysaccharide treatment (*N* = 3). **(B)** Role of GR and mineralocorticoid receptor using specific antagonists, such as mifepristone and spironolactone, respectively, on low concentration CORT preexposure effects on IL-6 release from BV2 cells (*N* = 5). Error bars represent mean ± SEM. Asterisks denote *p*-values *<0.05, **<0.01, ***<0.001, and ****<0.0001.

Corticosterone, at concentrations equal to and above 150 nM exhibited inhibitory effects on IL-6 regardless of preexposure or co-treatment with LPS (Figure 3A). A linear model constructed significantly explained variability in LPS-induced (LPS-vehicle treatment) supernatant IL-6 measured from BV2 cells after 24 h CORT preexposure or co-treatment, represented as a percentage of each corresponding vehicle pretreatment condition (*R*^2^ = 0.74, *p* < 0.0001). The model revealed significant CORT pretreatment inhibition of LPS-induced IL-6 at 150 nM [preexposure: *B* = −24.41%, *t*(19) = −2.10, *p* < 0.05; co-treatment: *B* = −37.57%, *t*(19) = −2.64, *p* < 0.05], 300 nM [preexposure: *B* = −25.28%, *t*(19) = −2.17, *p* < 0.05; co-treatment: *B* = −48.73%, *t*(19) = −3.42, *p* < 0.01], 500 nM [preexposure: *B* = −56.46%, *t*(19) = −4.86, *p* < 0.001; co-treatment: *B* = −48.20%, *t*(19) = −3.39, *p* < 0.01], and 1 µM [preexposure: *B* = −35.53%, *t*(19) = −3.06, *p* < 0.01; co-treatment: *B* = −57.01%, *t*(19) = −4.00, *p* < 0.001] concentrations. Pairwise comparisons between least squares means for each group using Tukey’s adjustment did not show any significant group differences between each concentration regardless of preexposure or co-treatment models (*p* > 0.05). There was no significant independent effect of pretreatment model [*B* = 5.64%, *t*(19) = 0.34, *p* = 0.74].

A coadministration of GR antagonist, mifepristone, abolished CORT pretreatment induced inhibition of IL-6 [*B* = 9.6 ng/ml, *t*(20) = 3.34, *p* < 0.01] (Figure 3B). Co-treatment model also resulted in increased IL-6 inhibition overall [*B* = −16.5 ng/ml, *t*(20) = −5.73, *p* < 0.0001]. *Post hoc* pairwise comparisons further revealed that mifepristone significantly reversed CORT preexposure (mean difference = 9.6 ng/ml, *p* < 0.01) and co-treatment (mean difference = 26.1 ng/ml, *p* < 0.0001)-induced inhibition. An MR antagonist did not modify CORT preexposure (mean difference = 3.2 ng/ml, *p* = 0.52) but significantly attenuated CORT co-treatment (mean difference = 7.7 ng/ml, *p* < 0.05) inhibition of IL-6 release from BV2 cells.

### Low Concentration CORT Preexposure Enhances LPS-Induced IL-1β Release when Controlling for Intracellular Pro-IL-1β Expression

**Figure 4:**
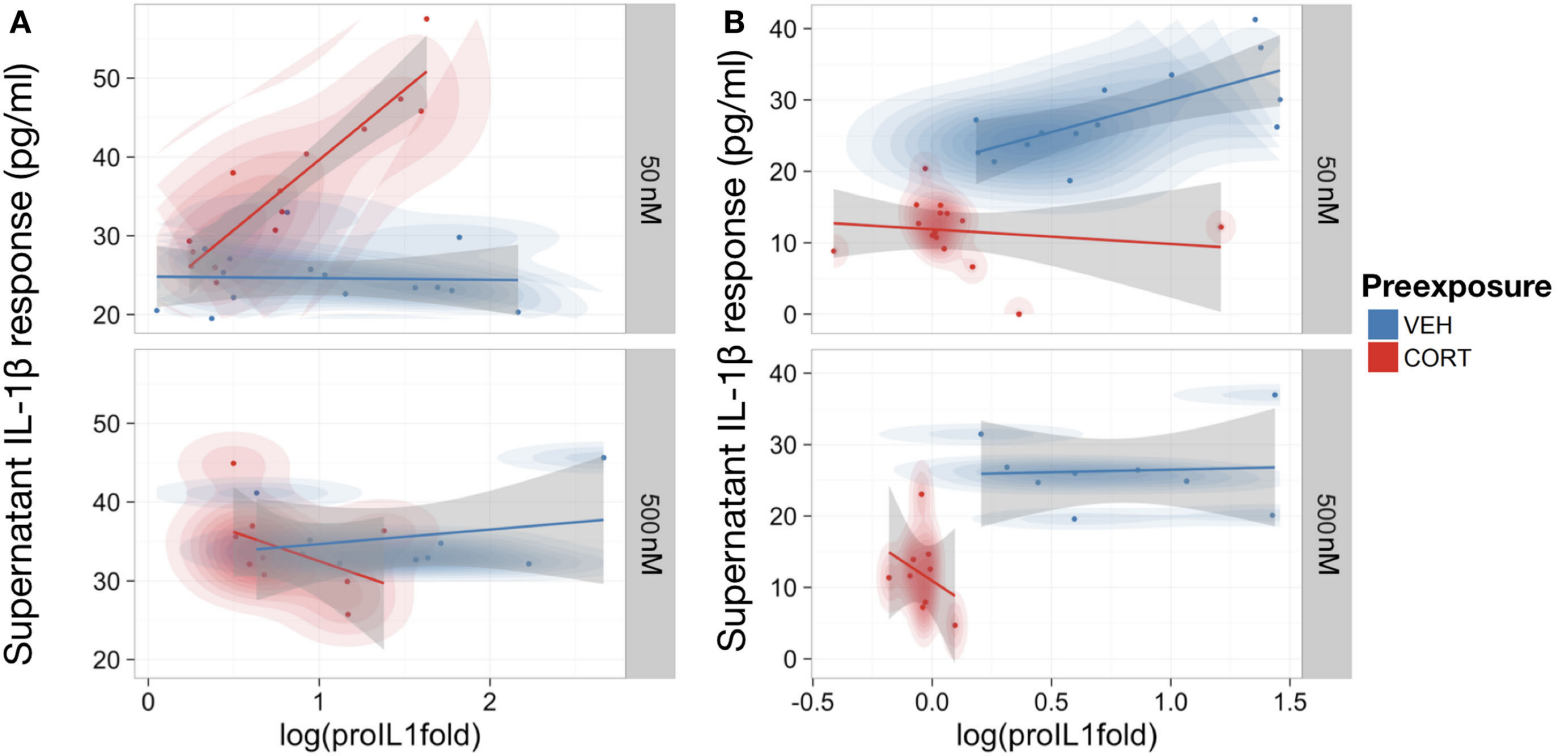
Low concentration corticosterone (CORT) preexposure increased release of supernatant IL-1β, but high concentration CORT preexposure and CORT co-treatment decreased release of supernatant IL-1β and cell expression of pro-IL-1β protein in lipopolysaccharide (LPS)-stimulated BV2 cells. **(A,B)** Scatter plot with probability density illustrating the linear-mixed effects model constructed between intracellular pro-IL-1β protein expression and supernatant IL-1β levels after 100 ng/ml LPS treatment following low (50 nM; *n* = 16) and high (500 nM; *n* = 8) concentration CORT vs vehicle preexposed cells. Preexposure model **(A)** and CORT co-treatment model **(B)**. All supernatant IL-1β values reported subtracted vehicle (no LPS)-treated baseline levels as control.

As IL-1β requires conversion from pro-IL-1β to mature IL-1β within the cell before release (36), the relationship between intracellular pro-IL-1β and supernatant IL-1β levels was investigated to determine if CORT had disrupted this process. An LME model was used to control for repeated measures from the same passage of cells, assessing the relationship between extracellular IL-1β and intracellular pro-IL-1β expression measured in pretreatment models (Figure 4A).

Corticosterone co-treatment with LPS inhibited IL-1β release (mean difference and 95% CI for CORT present–not present during LPS treatment = −11.36 ± 7.06 pg/ml). Therefore, CORT attenuates LPS-induced IL-1β secretion when present during LPS treatment.

While there was no significant overall relationship between pro-IL-1β and supernatant IL-1β in vehicle preexposure [*B* = 4.14, *t*(27) = 1.22, *p* = 0.23], CORT preexposure caused a significantly more positive relationship between pro-IL-1β and released IL-1β [*B* = 19.56, *t*(27) = −4.47, *p* < 0.001]. Importantly, there was also a significant interaction between CORT preexposure, preexposure concentration, and pro-IL-1β [*B* = 26.78, *t*(27) = 4.01, *p* < 0.001], indicating that low concentration CORT preexposure significantly potentiates the conversion and release of IL-1β in response to LPS, with no influence on the total pro-IL-1β expression levels (mean difference and 95% CI for LPS-treated vehicle preexposed–LPS-treated CORT preexposed cells = 0.34 ± 0.18). In CORT co-treated BV2 cells, there was an overall positive relationship between pro-IL-1β expression levels and supernatant IL-1β levels [*B* = 18.57, *t*(26) = 5.36, *p* < 0.01], but CORT co-treatment did not significantly modify this relationship [*B* = −8.40, *t*(26) = 0.82, *p* = 0.42] (Figure 4B).

### Low Concentration CORT Preexposure Enhances NF-κB Translocation in BV2 Cells *via* MR

**Figure 5:**
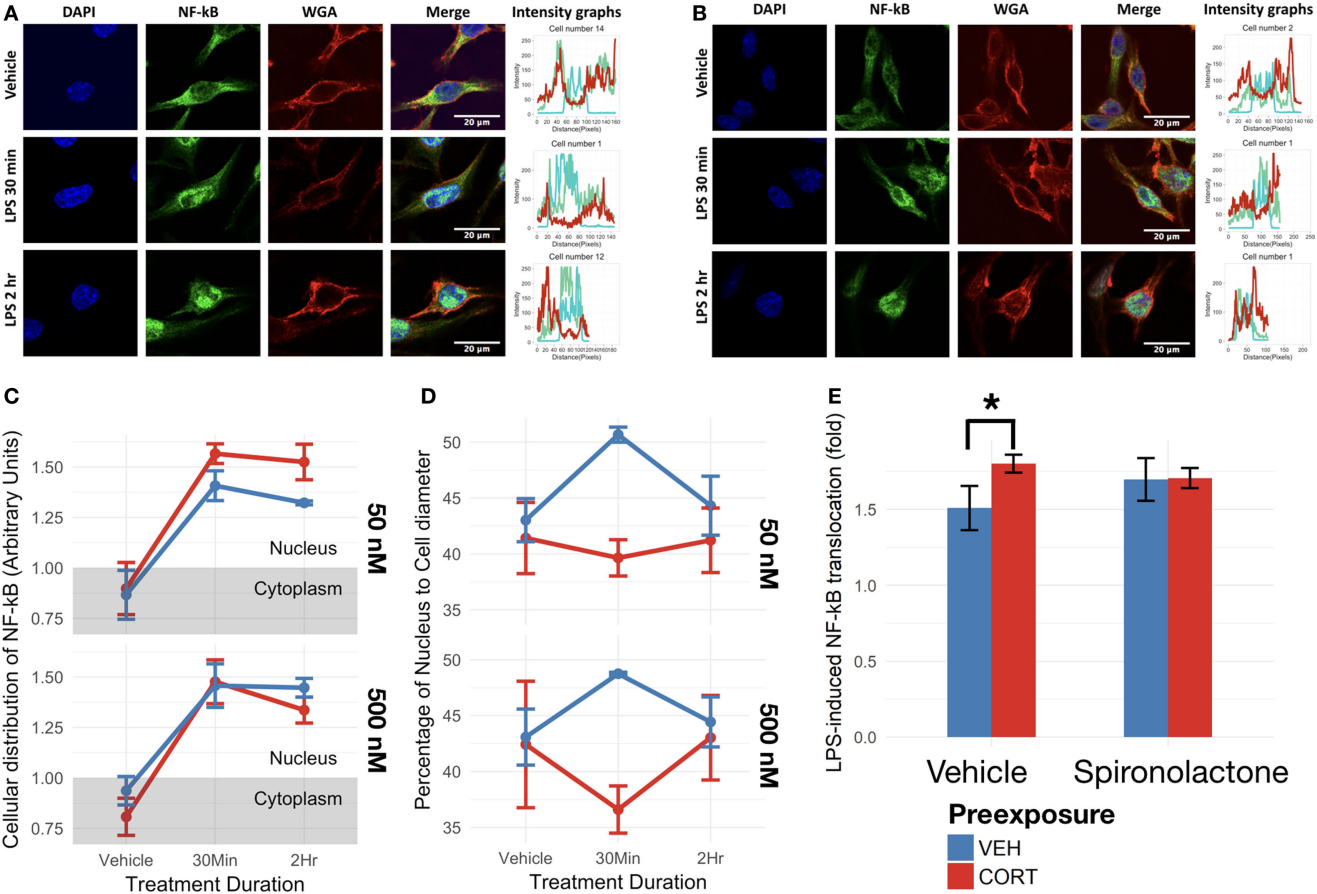
Corticosterone (CORT) preexposure (50 nM) increased NF-κB translocation, while both 50 and 500 nM prevented increased nucleus:cell diameter 30 min after lipopolysaccharide (LPS) treatment. **(A,B)** Fluorescent immunocytochemistry for NF-κB (green), nucleus (blue), cell membrane (red), and profile intensity plots across cell diameter shown for one sample cell in vehicle vs 30 min vs 2 h LPS following: **(A)** vehicle and **(B)** low concentration (50 nM) CORT preexposure. **(C)** Summary values representing nuclear expression of NF-κB proportional to total expression of NF-κB across the cell diameter, further normalized to the proportion of nucleus to cell diameter. Value <1: expression distribution favors cytoplasm, value >1: expression distribution favors nucleus. **(D)** Summary values for nucleus:cell diameter at baseline, 30 min and 2 h following LPS treatment. A lower value reflects decreased nucleus proportion of cell diameter, a measure of increased process length. **(E)** Effect of mineralocorticoid receptor inhibition *via* spironolactone treatment on LPS-induced NF-κB translocation (*N* = 5). All measures were made after preexposure + treatment. Error bars represent mean ± SEM values. Asterisks denote *p*-values *<0.05, **<0.01, ***<0.001, and ****<0.0001.

Given that low concentration CORT preexposure primed both LPS and TNF-α-induced IL-1β responses, increased NF-κB translocation may a potential mechanism of this effect. The effect of CORT preexposure on LPS-induced NF-κB translocation was thus investigated (Figure 5).

As expected, LPS treatment significantly upregulated BV2 cell NF-κB p65 translocation in both low [*F*(1,13) = 59.33, *p* < 0.0001] and high CORT concentration preexposure conditions [*F*(1,13) = 62.02, *p* < 0.0001] (Figures 5A,B). Low concentration CORT preexposure in BV2 cells significantly increases NF-κB translocation [*F*(1,13) = 4.89, *p* < 0.05]. However, no significant interaction between low concentration CORT preexposure and LPS treatment was observed on NF-κB translocation [*F*(1,13) = 1.13, *p* = 0.31]. No significant preexposure [*F*(1,13) = 0.58, *p* = 0.46], nor preexposure × treatment interactions [*F*(1,13) = 0.35, *p* = 0.56] were observed in high concentration preexposure conditions in BV2 cells (Figure 5C). Tukey’s *post hoc* test did not show any significant difference between individual groups (*p* > 0.05).

To verify if CORT-induced increase in NF-κB translocation was dependent on MR, spironolactone was coadministered during the preexposure phase (Figure 5E). In this experiment, CORT independently resulted in elevated LPS-induced NF-κB translocation compared with vehicle controls [*B* = 0.29-fold, *t*(10) = 2.87, *p* < 0.05], while spironolactone alone did not significantly influence NF-κB translocation [*B* = 0.19-fold, *t*(10) = 1.84, *p* = 0.10]. Furthermore, spironolactone and CORT co-treated cells did not exhibit CORT-induced increase in NF-κB translocation (mean difference = 0.090-fold, *p* = 0.93).

### CORT Preexposure Induced a Significantly More Ramified Morphology in Response to LPS Treatment

Measuring the length of the widest BV2 cell diameter and the proportion of nucleus to total cell diameter provides a measure of cell morphology. In this case, a higher value indicates an ameboid BV2 cell shape, and a lower value indicates a ramified BV2 cell morphology.

At both low and high concentrations, CORT preexposure induced a significantly more ramified morphology in BV2 cells overall [*F*(1,27) = 7.84, *p* < 0.01] (Figure 5D). In addition, the two-way ANOVA analysis revealed a significant interaction effect between LPS treatment duration and preexposure [*F*(1,27) = 5.33, *p* < 0.05]. *Post hoc* analysis conducted using Tukey’s correction further revealed a significantly lower nucleus:cell diameter in CORT + LPS conditions compared with vehicle preexposure + LPS conditions overall (mean difference = 6.39, *p* < 0.05), and CORT + LPS 30 min conditions compared with vehicle + LPS 30 min conditions (mean difference = 9.68, *p* < 0.05).

## Discussion

The current results have provided evidence that CORT can cause a non-classical change in innate-immune responses in BV2 microglia-like cells. Low concentration (50 nM) 24 h CORT preexposure resulted in increased NF-κB p65 translocation while preventing amoeboid cell morphology in response to LPS exposure. Furthermore, low concentration CORT preexposure preferentially sensitized the IL-1β response to LPS in an MR-dependent manner. Conversely, IL-6 responses were suppressed by both CORT preexposure and co-treatment, without priming at the low CORT concentrations. These immune-suppressive actions on IL-1β and IL-6 were further shown to be mediated by GR activity. To the best of our knowledge, this is the first study to show the mechanisms underpinning direct CORT priming and immunosuppressive actions on BV2 microglia-like cells *in vitro*, providing a basis to further investigate the neuroimmune system as a potential mediator of stress-induced maladaptations.

This study found that low concentrations of CORT in BV2 cells induces an inflammatory profile that is characterized by increased NF-κB nuclear translocation and IL-1β release, while retaining ramified morphology during LPS challenges as compared with vehicle preexposed cells. This is consistent with previous findings showing that stress can induce increased ramified morphology in prefrontal microglia (37). We thus propose that CORT preexposure can prime BV2 cells toward a distinct secretory and gene expression profile while retaining a ramified cell morphology, which is indicative of “resting” state (32). These results suggest that CORT preexposure may prime a pro-inflammatory state, while protecting BV2 cells from overresponding to immune stimulus.

Corticosterone, being a predominantly anti-inflammatory steroid hormone, was shown to suppress IL-1β and IL-6 secretion from LPS-treated cells when present during the subsequent immune challenge. This is consistent with the classical research (17, 38) and indicates the robustness of the anti-inflammatory effects of CORT. However, concentration response results obtained indicate that low concentration CORT preexposure primed IL-1β release following LPS stimulation.

Interestingly, this CORT preexposure priming effect was conserved in TNF-α-induced IL-1β release (Supplementary Material). Together with the increased NF-κB translocation in BV2 cells preexposed to low concentration CORT, these results suggest that CORT may cause IL-1β release *via* adaptations to NF-κB signaling. IL-1β is a potent pro-inflammatory cytokine, which is implicated in various neurological disease states from neuronal hyperexcitability in epilepsy (39), to impairments in learning and memory, as well as perpetuation of sickness and depressive behaviors (40). This CORT-induced priming of IL-1β secretion could contribute to the maladaptive outcomes of stress.

Although the elevation of IL-1β only occurs in lower concentrations of CORT preexposure, this effect is supported by previous studies (41, 42). Smyth et al. (41) established that 100 nM CORT could prime future immune responses in RAW264.7 macrophage-like cells after removal of CORT. This low concentration effect was further explored by Yeager et al. (43). Similar to the findings here, the researchers described a biphasic concentration response to cortisol in humans, where patients who received cortisol preexposure exhibited exaggerated responses to LPS challenge in a concentration- and time-dependent manner. CORT has also been shown to exhibit permissive effects toward inflammation at low concentrations, inhibitory at higher concentrations, and mediate the priming effects in inflammation following stress (14, 21, 44). Therefore, the specific circumstances that allow CORT to cause a switch from anti- to pro-inflammatory actions on IL-1β after removal from the system is consistent with the hypothesis that stress can cause immune sensitization during the “recuperation phase” (15).

Using specific antagonists to GR and MR, the two CORT-binding receptors, this study has further demonstrated that this low concentration CORT priming of NF-κB translocation and IL-1β release is MR dependent, while high concentration CORT-induced suppression of IL-1β relies on GR binding. This result is consistent with the binding properties of the receptors implicated in the immune-priming and immune-suppressive actions of CORT; as MR is a high-affinity and low abundance receptor for CORT, while GR is a low-affinity and high-abundance receptor (45). Moreover, previous findings showed that both CORT and aldosterone, an MR-specific agonist, was also able to increase NF-κB translocation and downstream pro-inflammatory gene transcription in BV2 cells (22). The authors were also able to inhibit this increase in NF-κB translocation using an MR antagonist, thus consistent with the MR-dependent CORT priming of pro-inflammatory responses seen here. The removal-dependent preexposure of CORT demonstrated here therefore fits into the paradigm of priming innate-immune responses following small increases in CORT concentration and further provided evidence that this effect may be related to MR rather than GR actions. This result highlights the mechanistic importance of MR in glucocorticoid actions on immunocompetent cells.

In addition, the lack of GR dependency in the current findings indicates that glucocorticoid resistance, a GR-mediated effect (25), unlikely participates in the priming responses seen from low concentration CORT preexposure models. In support of our findings of MR-mediated priming, MR appears to be important toward pro-inflammatory responsivity of on immunocompetent cells, as evidenced by reduced macrophage migration, pro-inflammatory gene expression and proliferation in MR-deficient mice (46). However, the exact mechanisms underpinning the pro-inflammatory actions of MR are currently unclear. Some evidence of MR interactions with NF-κB transcriptional factor *via* p38MAPK phosphorylation has previously been found in vascular smooth muscle cells (47), but this has not been verified in immunocompetent cells. Elucidating the exact mechanism of MR-mediated priming of pro-inflammatory responses is therefore an important pursuit in future studies.

Despite finding pro-inflammatory priming effects of CORT on IL-1β and NF-κB translocation, other results here demonstrate the complexity in this effect. First, unlike IL-1β, CORT exhibited a dose-dependent single-phase suppression of LPS-induced IL-6 responses from the same cells, regardless of preexposure or co-treatment. Interestingly, 150 nM CORT preexposure elicited both priming of IL-1β and inhibition of IL-6 responses. Furthermore, the inhibition of IL-6 responses was reversed by GR antagonism, suggesting a fine balance between GR and MR signaling in the preexposure model used here. Taken together, the inhibition of IL-6 signaling seen here supports the classical view of CORT immunosuppression *via* GR actions (48), but this may not occur in isolation of priming effects on IL-1β from the same cells.

Second, the current results showed no significant effect of CORT on NLRP3 expression, while the 24 h LPS-induced IL-1β release seen here was not caspase-1 dependent (Supplementary Material). Thus, the effects observed in the current model involve an inflammasome-independent component. In support of the current findings, primary microglia have previously demonstrated reduced caspase-1 dependency (49), suggesting that the lack of IL-1β attenuation may be related to cell type. In addition, low concentration CORT preexposure did not cause an increase in cytotoxicity (Supplementary Material). The elevation of IL-1β seen here is therefore unlikely due to cell death or damage. Further analysis of gene expression correlates with IL-1β protein release identified differing contributing gene expressions in CORT preexposed cells when compared with vehicle preexposure (Supplementary Material). Together with the lack of IL-6 priming, these findings suggest that rather than amplifying all LPS-induced pro-inflammatory responses, CORT preexposure effects appear to involve complex adaptations to the pro-inflammatory signaling pathways. To investigate these complex adaptations, further study of genomic and non-genomic actions of glucocorticoid signaling *via* both GR and MR in the context of immune priming is therefore required.

An important consideration is the use of BV2 cells in this study, an immortalized cell line developed from mouse microglia using retroviral infection (50). BV2 cells have impaired IRF3-dependent gene transcription following LPS treatment when compared with primary microglia harvested from neonatal mice, indicating altered TRIF signaling (51). Despite these differences, morphological change (31), NLRP3 inflammasome activation (52), TLR4-MyD88 signaling (53), and NF-κB translocation (54), have all been shown similarly in BV2 cells and primary microglia. BV2 cells are therefore immunocompetent and bear functional similarities to primary microglia. However, primary microglial cell culture should still be pursued in future studies for translation of these findings to age- and sex-related differences in stress models.

## Conclusion

This study found that CORT, a predominantly anti-inflammatory steroid hormone, is also able to produce non-classical sensitized pro-inflammatory responses in BV2 microglia-like cells. The MR-dependent pro-inflammatory effect of CORT is apparent after low concentration 24 h preexposure, after which increases in NF-κB p65 nuclear translocation, and ultimately IL-1β conversion and release, can be detected following LPS treatment. Thus, low concentration CORT potentially primes responses in BV2 microglia-like cells to a subsequent innate-immune challenge.

IL-1β may further provide a direct link between the neuroendocrine stress response and depressive behaviors. For example, chronically stressed mice with small elevations in circulating CORT also have increased circulating and brain IL-1β expression, concurrent with increased anhedonia and decreased social exploration, all measures of rodent depressive-like behaviors (55). In the same study, IL-1 receptor knockout mice and adrenalectomized wild-type mice exhibit similar protection from behavioral effects of chronic stress, providing some evidence that IL-1β and CORT signaling may both be involved. The current findings provide evidence that, under the right conditions, CORT can directly influence microglia-like cells to favor secretion of IL-1β in the event of TLR4 activation, thus identifying a link between the two systems in innate-immune function.

## Author Contributions

Experimental design and preparation of manuscript was done by JL. SM, DB, and MH provided support in experimental methods and editing of manuscript. MH and SM oversaw the proceedings of this project.

## Conflict of Interest Statement

The authors declare that the research was conducted in the absence of any commercial or financial relationships that could be construed as a potential conflict of interest.
