# Supplementary material for: Corticosterone Preexposure Increases NF-κB Translocation and Sensitizes IL-1β Responses in BV2 Microglia-Like Cells
Source: Front Immunol. 2018 Jan 22;9:3. doi: 10.3389/fimmu.2018.00003 (PMC5786551; doi:10.3389/fimmu.2018.00003)
Supplement: Supplementary file 1 [file Data_Sheet_1.PDF]

Supplementary methods

Polymerase Chain Reaction

To investigate steroid receptor, TLR4 pathway, and inflammasome-related gene transcription following a 6 h and 24 h inflammatory challenge (100 ng/ml LPS), real time quantitative PCR was conducted according to manufacturer's specifications (Biorad 175271). RNA was extracted from BV2 cell samples using a Maxwell 16 LEV simplyRNA Cells kit (Promega A208B) according to manufacturer's recommendations. RNA concentration and purity were determined by UV absorbance at 260 and 280 nm. cDNA templates were obtained via reverse transcription (High Capacity cDNA reverse transcription kit [Applied Biosystems 4638814]) of 900ng of RNA according to manufacturer standard protocol. Triplicate PCR reactions (40 cycles [95 °C for 5 s -> 62°C for 30 s]) containing 10 uL of cDNA, 300 nM primers (Table 1.) and 10 uL SYBRgreen iTaq Supermix (Biorad 1725120) in a final volume of 20 uL detected Gapdh, Nr3C1, Nr3c2, Trif, Md2, Cd14, Tlr4, MyD88, Nlrp3, Pycard and Casp1. SYBRgreen fluorescence signal was measured using the Biorad CFX96 thermocycler. Reaction efficiencies between 90-105% were confirmed, and linear dynamic ranges determined, for each gene using 5 serial dilutions of cDNA from LPS and vehicle-treated BV2 cells.

Fold change was quantified as $2^{-\Delta\Delta ct}$ from Gapdh and a standard un-treated control sample. Product specificity was confirmed by post-PCR melt-curve analysis. Absence of dsDNA amplification was confirmed by a No Reverse Transcription control, and no template (water) controls were included in every gene assay run to control for non-specific PCR amplification.

Primer	Group	Forward	Reverse
GAPDH	CONTROL	AGG TCG GTG TGA ACG GAT TTG	TGT AGA CCA TGT AGT TGA GGT CA
NR3C1	STEROID RECEPTOR	AGC TCC CCC TGG TAG AGA C	GGT GAA GAC GCA GAA ACC TTG
NR3C2	STEROID RECEPTOR	GAA GAG CCC CTC TGT TTG CAG	TCC TTG AGT GAT GGG ACT GTG
TRIF	TLR PATHWAY	AAC CTC CAC ATC CCC TGT TTT	GCC CTG GCA TGG ATA ACC A
MD2	TLR PATHWAY	CGC TGC TTT CTC CCA TAT TGA	CCT CAG TCT TAT GCA GGG TTC A
CD14	TLR PATHWAY	CTC TGT CCT TAA AGGC GGC TTA C	GTT GCG GAG GTT CAA GAT GTT
TLR4	TLR PATHWAY	GCC TTT CAG GGA ATT AAG CTC C	GAT CAA CCG ATG GAC GTG TAA A
MYD88	TLR PATHWAY	TCA GTG TCT TAC CCT TGG T	AAA CTG CGA GTG GGG TCA G
NLRP3	INFLAMMASOME	ATT ACC CGC CCG AGA AAG G	TCG CAG TCA AAG ATC CAC ACA G
Pycard	INFLAMMASOME	CTT GTC AGG GGA TGA ACT CAA AA	GCC ATA CGA CTC CAG ATA GTA GC
CASP1	INFLAMMASOME	ACA AGG CAC GGG ACC TAT G	TCC CAG TCA GTC CTG GAA ATG

Table 1. Primer sequences and gene grouping used.

Statistics

Effects of pre-exposure conditions on gene transcription fold change of LPS-treated/ vehicle-treated cells were analysed by multiple linear regression, with variable selection and regularisation via LASSO regression and cross-validation analysis using the glmnet statistical package. Further analysis on individual gene contribution towards the resultant linear regression model after regularisation was obtained via relaimpo statistical package.

To quantify effects of treatment (LPS vs Vehicle), pre-treatment drug (CORT vs Vehicle), pre-treatment concentration (50 nM vs 500 nM) and pre-treatment model (pre-exposure vs co-treatment), a 4-way ANOVA followed by post-hoc pairwise comparisons using Tukey’s correction were applied to evaluate total intracellular NLRP3 expression.

Supplementary results

Low concentration CORT does not significantly modify cytotoxicity

Cytotoxicity, as measured by LDH activity measured from cell supernatants following 24 h 50 nM CORT or vehicle exposure, was not significantly influenced by CORT ($t(10) = -0.32$, $p = 0.76$).

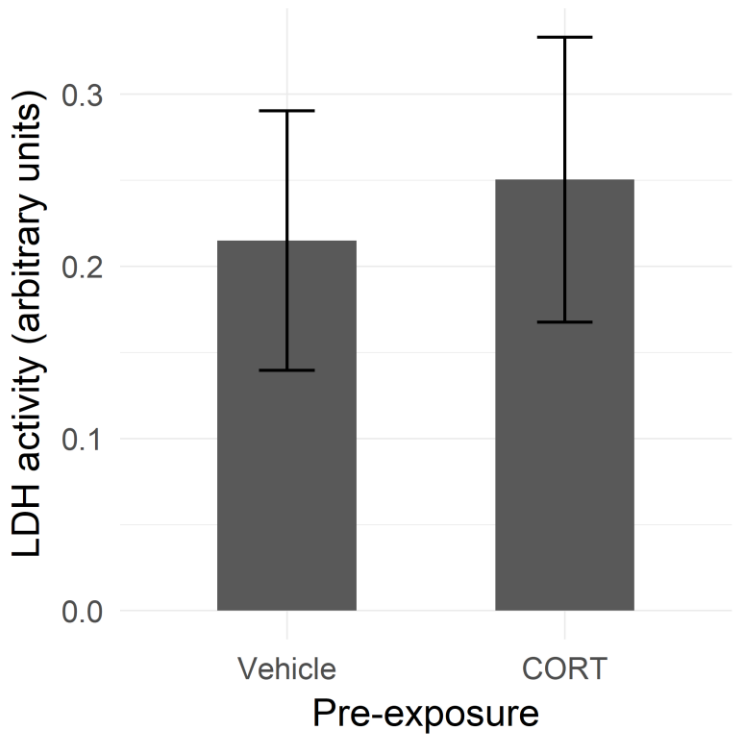

Supplementary figure 1. Low concentration CORT does not significantly influence LDH activity. LDH activity measured from supernatants following 24 h 50 nM CORT or vehicle treatment (N= 6). Bar graphs represent mean ± sem of LDH activity.

Caspase inhibition fails to prevent IL-1β release from BV2 cells.

To test caspase-dependence of IL-1β release from BV2 cells, a cell permanent caspase-1 and caspase-4 inhibitor, z-YVAD-FMK (0, 100 nM, 1 µM, and 10 µM) was incubated in the presence or absence of LPS (100 ng/ml) for 24 h. Administration of z-YVAD-FMK *in vitro* was previously shown to inhibit IL-1β release (Halle *et al.*, 2008; Kaushik *et al.*, 2012), and Fas-mediated apoptosis (Choi, Jeong and Benveniste, 2004). In this experiment, extracellular IL-1β was measured after LPS treatment. This pilot experiment was done without CORT pre-exposure to characterise the concentrations required to achieve inhibition of IL-1β release (Supplementary figure 2).

As assessed by a linear mixed effects model controlling for repeated measures, YVAD-FMK did not significantly influence IL-1β release from BV2 cells at any concentration (100 nM: $B= 0.32$ fold, $t(21)= 0.65$, $p=0.52$; 1000 nM: $B=0.32$ fold, $t(21)= 0.65$, $p=0.52$, 10 uM: $B=-0.15$ fold, $t(21)=-0.30$, $p=0.76$). Thus, IL-1β release appears to be caspase-1 and caspase-4 independent in BV2 cells.

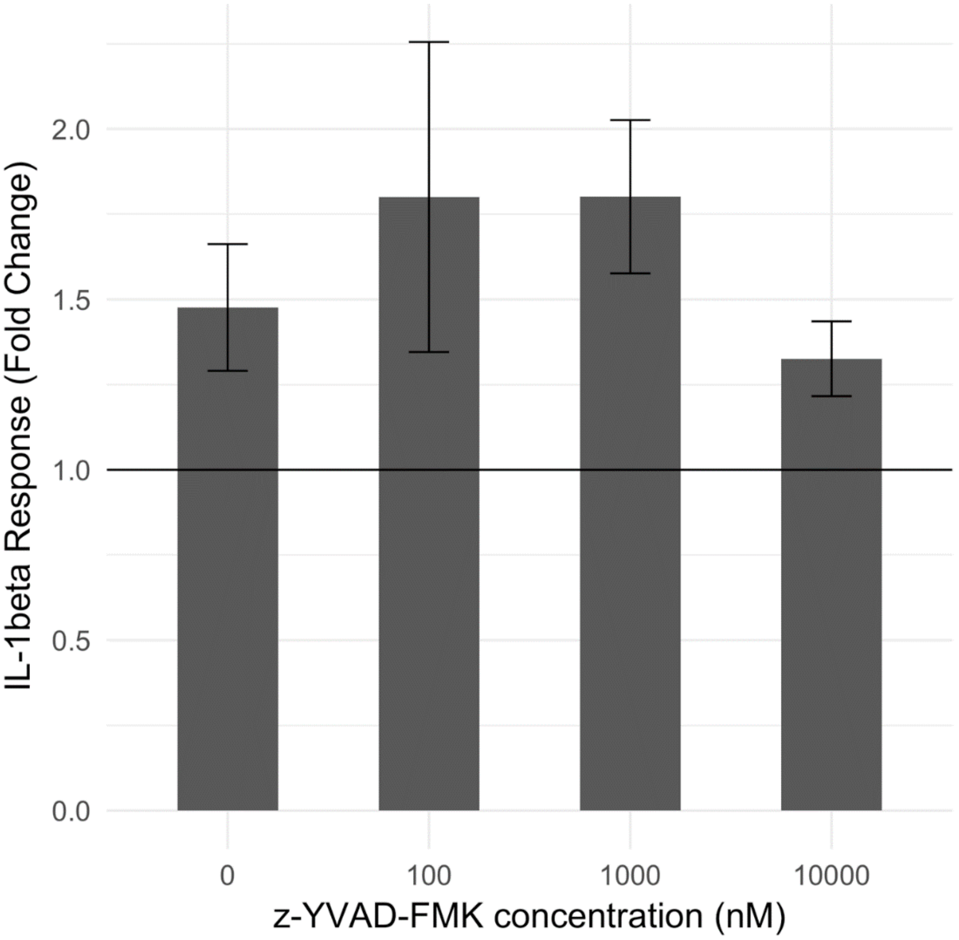

Supplementary figure 2. Pharmacological caspase-1 inhibition does not attenuate 24 h LPS-induced IL-1beta release from BV2 cells. A co-treatment of caspase-1 and caspase-4 inhibitor, z-YVAD-FMK (0, 100, 1000 and 10000 nM) with LPS (100 ng/ml) did not significantly attenuate IL-1β release. Bar graphs represent mean mean± SEM of IL-1β fold change (N=4).

CORT pre-exposure in BV2 cells does not alter total NLRP3 protein expression

NLRP3 constitutes part of the inflammasome complex which cleaves pro-IL-1β into mature IL-1β before release from microglia (Hanamsagar et al., 2011). Protein levels of NLRP3 were thus measured to assess if CORT pre-exposure may be altering inflammasome expression. NLRP3 staining using fluorescent immunocytochemistry confirmed the presence of NLRP3 in the cytosol of BV2 cells following LPS treatment (supplementary figure 3A, 3B).

Western blot quantification of NLRP3 expression showed that LPS treatment ($F(1, 191)= 55.41$, $p < 0.0001$) significantly increased NLRP3 expression (supplementary figure 3C). High concentration pre-exposure, regardless of volume-matched ethanol vehicle or CORT, exhibited a significantly lower NLRP3 expression versus low concentration pre-treatments overall ($F(1,191) = 14.11$, $p< 0.001$), whereas co-treatment of LPS and CORT or vehicle resulted in more NLRP3 protein expression compared to pre-treated cells ($F(1,191) = 4.36$, $p< 0.05$). However, CORT pre-treatment in itself did not significantly influence total NLRP3 expression ($F(1,191) = 0.036$, $p = 0.85$; Fig. 4A). These data suggest that the CORT-mediated increased conversion and subsequent release of IL-1β with LPS is not due to increases in total NLRP3 expression within the cytoplasm.

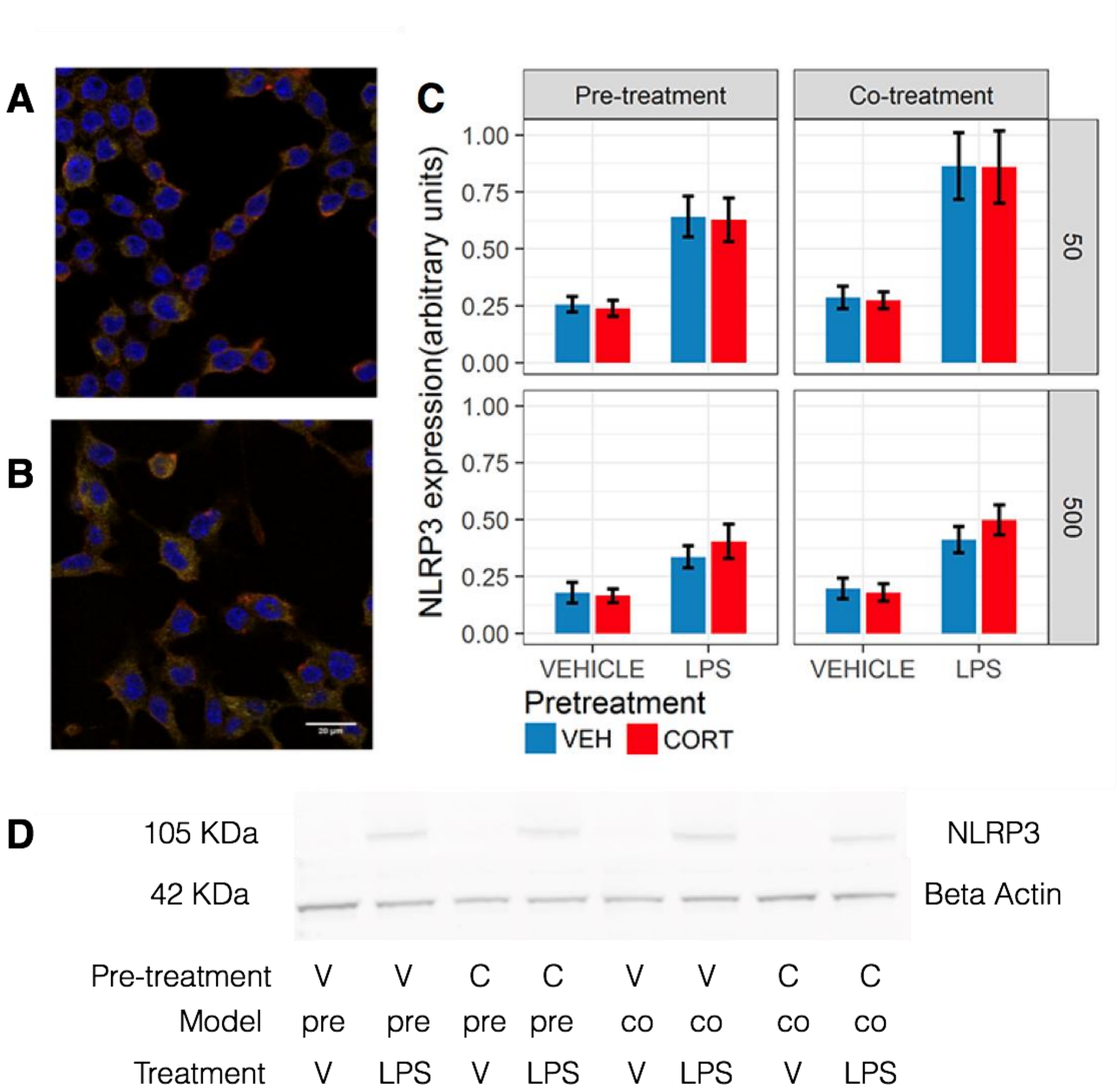

Supplementary Figure 3. CORT pre-exposure and co-treatment do not alter NLRP3 expression following LPS. A,B) Fluorescent Immunocytochemistry showing NLRP3 expression (yellow) in the cytoplasm, between nucleus (Blue) and cell membrane (Red) in CORT (B) and Vehicle (C) pre-exposed cells and LPS treatment. C) Western blot measurements of NLRP3 expression measured from BV2 cells that received either Low (50 nM; n = 16) or High (500 nM; n =8) concentration CORT or vehicle exposure, followed by LPS (100 ng/ml) or vehicle treatment. CORT pre-treatment included pre-exposure and co-treatment conditions. D) Representative western blot displaying NLRP3 and Beta Actin protein expression for lysates of low concentration pre-treatment + treatment conditions.

Inflammasome-related and steroid receptor-related gene expression in CORT pre-exposed BV2 cells following 6 h and 24 h LPS administration:

The increase in NF-kB translocation in cells pre-exposed to low concentration CORT suggests that gene transcription may be altered. To identify possible contributing transcriptional correlates with IL-1β release following low concentration CORT pre-exposure, gene expression and secreted IL-1β protein were investigated in BV2 cells. Thus, concurrent TLR-related, inflammasome-related and steroid receptor-related gene expressions were measured via qPCR to investigate gene expression contribution towards CORT-induced priming of IL-1β protein secretion in BV2 cells (Figure 6A). A general negative relationship was found between most gene expression measures and IL-1β release, however, this is likely attributable to differences in the timing of peak transcription relative to protein release. Peak gene transcription was found at 6 h post LPS treatment, but IL-1β release was highest at 24 h post LPS.

Due to a relatively large number of gene expression measures, there was redundancy within the model. Lasso regularisation was therefore utilised to identify key genes contributing to the variation in IL-1β release in response to LPS, and yielded different sets of contributing genes for vehicle (R^2 = 0.61; Tlr4, Cd14, Nr3c2 and Nlrp3), and CORT (R^2 = 0.94, Pycard, Nlrp3 and Cd14) pre-exposed BV2 cells (Figure 6A). Furthermore, relative importance analysis applied to each model revealed varying contributions for each gene in both pre-exposure conditions (Figure 6B). In vehicle pre-exposed cells, Nlrp3 accounted for 61.1% of 61.4% total variation explained in the linear model. In CORT pre-exposed cells however, Nlrp3 mRNA expression only accounted for 37.1% of 93.9% variation in IL-1β, whereas Pycard

and Cd14 mRNA expression contributed 31.1% and 29.3% respectively, thus indicating that CORT pre-exposure has an impact on LPS-induced gene transcription in relation to IL-1β release from BV2 cells.

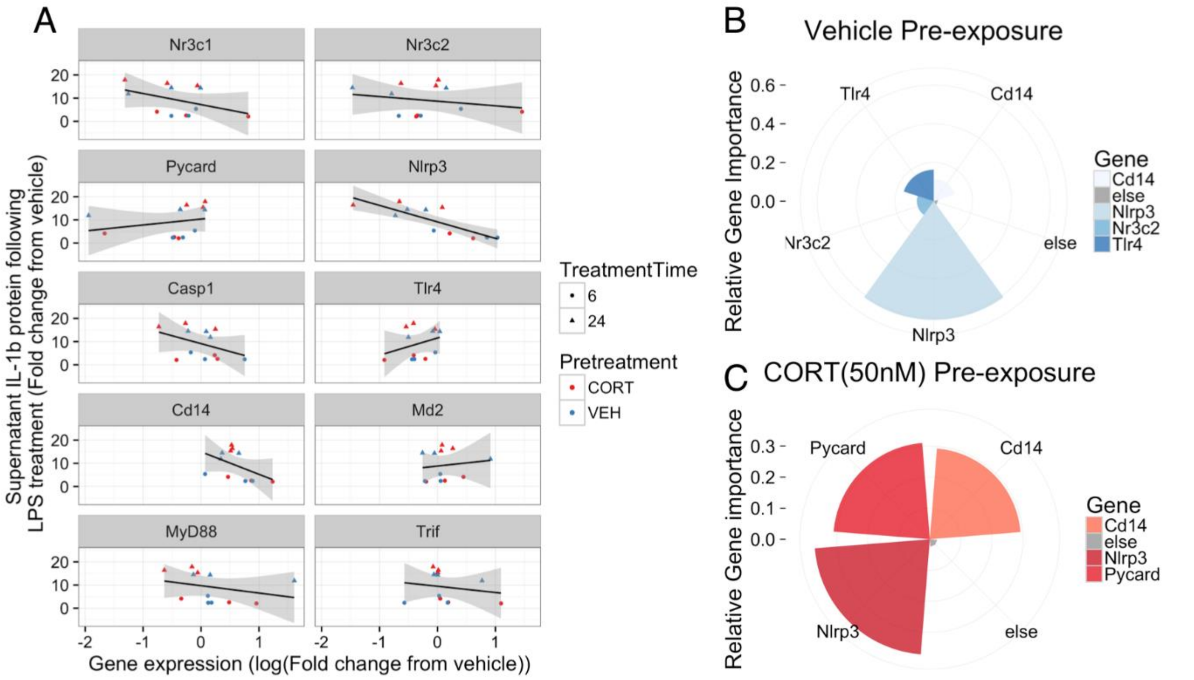

Supplementary Figure 4. Changes in IL-1β release in BV2 cells receiving CORT 50 nM pre-exposure is associated with increased involvement of Pycard and Cd14 gene expression profiles 6 h and 24 h post LPS administration. A) Individual gene expression correlates with LPS-induced IL-1β protein secretion measured in supernatant from BV2 cells, following either 50 nM CORT or volume-matched vehicle pre-exposure (N = 3). Gene expression is represented as fold change from vehicle/vehicle treated controls. B-C) Relative contribution of each lasso regularisation-selected gene in multiple linear regression models, explaining variation in LPS-induced IL-1β protein release in Vehicle pre-exposed (B) and 50 nM CORT pre-exposed (C) BV2 cells.

CORT pre-exposure sensitized TNF-α-induced IL-1β release from BV2 cells

LPS is a compound that exists on gram negative bacterial cell walls, which do not readily cross the blood brain barrier under normal circumstance (see review Coureuil et al., 2017). Instead, humoral mechanisms, via cytokine responses in the periphery have been proposed as main communicators between the peripheral immune system and microglia in the CNS (McCusker and Kelley, 2013). Thus, TNF-α (100ng/ml) was used in place of LPS during the treatment stage. TNF-α was selected as the immune stimulant since it is an early phase pro-inflammatory cytokine which stimulates IL-1β release from innate immune cells (Franchi et al., 2009).

Since all treatments and controls were applied to each individual biological replicate, a linear mixed effects model was used to account for repeated measures for each biological replicate. In this experiment, CORT pre-treatment significantly increased the TNF-α induced change in IL-1β release ($B = 0.17$ fold, $t(26)=4.35$, $p<0.001$), while co-treatment in general decreased TNF-α induced IL-1β release ($B=-0.61$ fold, $t(26)=-2.98$, $p<0.01$) (Figure 2E). CORT pre-treatment significantly interacted with co-treatment model to cause an even greater decrease in IL-1β ($B=-0.63$ fold, $t(26)=-2.15$, $p<0.05$). Post hoc pairwise group comparisons with Tukey's correction further showed that 50nM CORT pre-exposure (mean difference = 0.73 fold, $p<0.01$) and 500 nM CORT pre-exposure (mean difference = 1.0 fold, $p<0.0001$) both significantly elevated TNF-α induced IL-1β release compared to volume-matched vehicle pre-exposure. CORT co-treatment at both concentrations did not significantly inhibit TNF-α induced IL-1β release when compared to volume matched vehicle co-treatments ($p>0.05$).

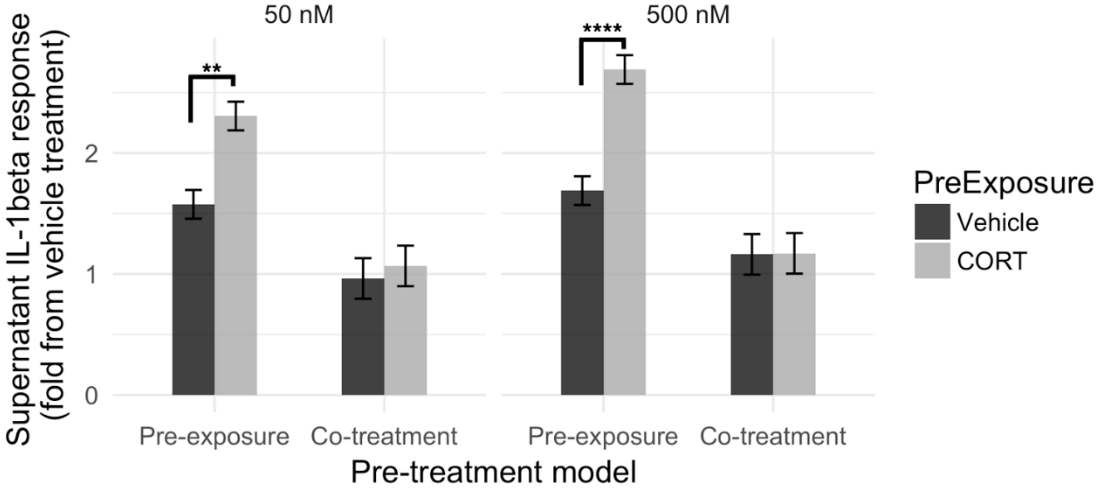

Supplementary figure 5. CORT pre-exposure increases TNF-α induced IL-1β release from BV2 cells. Fold change in IL-1β following 24 h pre-treatment + 24 h 100 ng/ml TNF-α. CORT pre-exposure increased IL-1β responses, while the co-treatment model resulted in no significant difference from vehicle pre-treated cells (N = 3). Error bars represent mean +- SEM. * <0.05, **<0.01, ***<0.001, ****<0.0001.
